# Doublecortin-like kinase 1 promotes stem cell-like properties through the Hippo-YAP pathway in prostate cancer

**DOI:** 10.7150/ijms.99062

**Published:** 2025-01-01

**Authors:** Donggen Jiang, Jun Li, He Ma, Binyuan Yan, Hanqi Lei

**Affiliations:** Department of Urology, Kidney and Urology Center, the Seventh Affiliated Hospital of Sun Yat-Sen University, Shenzhen, China.

**Keywords:** prostatic neoplasm, doublecortin-like kinase 1, Hippo, stemness

## Abstract

**Background:** Doublecortin-like kinase 1 (DCLK1) has been revealed to be involved in modulating cancer stemness and tumor progression, but its role in prostate cancer (PCa) remains obscure. Castration-resistant and metastatic PCa exhibit aggressive behaviors, and current therapeutic approaches have shown limited beneficial effects on the overall survival rate of patients with advanced PCa. This study aimed to investigate the biological role and potential molecular mechanism of DCLK1 in the progression of PCa.

**Methods:** The role of DCLK1 in maintaining PCa stem cell-like properties was explored via gain- and loss-of-function studies, including colony formation assays, sphere formation assays and measurement of stemness-related marker expression. A set of transcriptomic data for patients with PCa was downloaded from The Cancer Genome Atlas to analyze the correlations between DCLK1 and Hippo pathway gene expression. The mechanism by which DCLK1 regulates Hippo signaling and cancer stemness was further investigated *in vitro* by methods such as Western blot analysis, quantitative real-time PCR analysis, immunofluorescence staining, and luciferase reporter assays and *in vivo* by animal studies.

**Results:** The gain- and loss-of-function studies demonstrated that upregulating DCLK1 promoted but downregulating DCLK1 suppressed aspects of the PCa stem cell-like phenotype, including colony formation, sphere formation and the expression of stemness-related markers (c-Myc, OCT4, CD44, NANOG, SOX2, and KLF4). Importantly, bioinformatics analysis indicated that DCLK1 is closely correlated with the Hippo signaling pathway in PCa. Further *in vitro* assays revealed that DCLK1 inhibits the Hippo signaling pathway, leading to yes-associated protein (YAP) activation via large tumor suppressor homolog 1 (LATS1). Moreover, the effect of DCLK1 on abolishing stemness traits in PCa was observed after treatment with verteporfin, a small molecule inhibitor of YAP. Consistent with the *in vitro* findings, the *in vivo* findings confirmed that DCLK1 promoted the tumorigenicity and stem cell-like traits of PCa cells via Hippo-YAP signaling.

**Conclusion:** DCLK1 promotes stem cell-like characteristics by inducing LATS1-mediated YAP signaling activation, ultimately leading to PCa tumor growth and progression. Thus, our findings identify an attractive candidate for the development of cancer stem cell-targeted therapies to improve treatment outcomes in advanced PCa.

## Introduction

The second most frequently diagnosed malignancy in men, prostate cancer (PCa) is responsible for approximately 4.1% of cancer-related deaths worldwide, with an estimated 45 PCa-related deaths occurring per hour in 2022 1. When PCa is diagnosed at an advanced stage where radical therapy (prostatectomy or radiation) is no longer feasible, most patients gradually develop castration-resistant prostate cancer (CRPC) and eventually develop distant metastasis 2~3 years after initiation of androgen deprivation therapy (ADT), entering a fatal and terminal stage considered incurable 2. Therefore, further in-depth investigations are warranted to understand the mechanisms underlying disease progression, metastasis and drug resistance in PCa.

The recent identification of cancer stem cells (CSCs) provided new insights into the initiation, progression, and metastasis of numerous neoplasms, including PCa. CSCs are a unique subpopulation of cancer cells with traits similar to those of normal progenitor or stem cells within tumors and retain the capacity for multilineage differentiation and self-renewal to drive heterogeneity and tumor growth, and the acquisition of stem cell-like properties by cancer cells is a crucial step for cancer progression and treatment resistance 3, 4. Notably, the high resistance of these CSCs to different pharmacotherapies resulting from upregulation of drug resistance and antiapoptotic genes may also contribute to treatment failure, relapse and metastasis 3. Hence, revealing the molecular mechanisms underlying the regulation of stemness properties in PCa is essential for improving PCa treatment.

Doublecortin-like kinase 1 (DCLK1), a member of the doublecortin family and a serine/threonine protein kinase, was initially identified as a microtubule-associated protein expressed in the nervous system and involved in neurogenesis and neuronal migration 5. Recently, accumulating evidence has shown that DCLK1 plays critical roles in epithelial-mesenchymal transition, tumorigenesis, disease progression, and metastasis in numerous solid malignancies, including colorectal cancer, pancreatic adenocarcinoma, cholangiocarcinoma, and renal clear cell carcinoma 5-8. Importantly, published studies have also demonstrated that DCLK1 is essential for maintaining CSC-like phenotypes in several human cancers 7-9. We previously revealed that DCLK1 overexpression was correlated with PCa tumor aggressiveness and might effectively predict poor biochemical recurrence-free survival outcomes in patients after radical prostatectomy 10, but the related underlying molecular mechanism remains obscure. These published data, together with our preceding research results, intriguingly suggested that DCLK1 may promote PCa aggressiveness by regulating the stemness characteristics of PCa cells.

The Hippo-YAP pathway, which was originally identified by fly geneticists, is a centrally important conserved cascade supporting cancer cell survival that controls tumorigenesis in diverse cancer types by regulating cell proliferation, differentiation, apoptosis, and CSC traits 11, 12. Regarding the correlation between Hippo-YAP signaling and prostate cancer stem cell (PCSC) characteristics, Nong *et al.* 13 discovered that reduced death-associated protein kinase 1 expression might promote stem cell-like traits in PCa cells through activating zinc finger E-box-binding homeobox-1 via the Hippo/YAP signaling pathway. Importantly, by mechanistic studies, Liu *et al.* 14 demonstrated that inhibition of phosphodiesterase 5 activity attenuated colony formation, altered the expression patterns of stem cell markers, and increased cisplatin cytotoxicity, resulting in attenuation of stemness in PCSCs through Hippo signaling. Results obtained in both *in vitro* and *in vivo* models have similarly indicated that the combination of simvastatin and valproic acid can sensitize metastatic CRPC cells to docetaxel and reverse docetaxel resistance by targeting the PCSC compartment through YAP axis modulation 15. Furthermore, yes-associated protein 1 (YAP1) overexpression has been shown to contribute to the development of enzalutamide resistance by inducing the acquisition of cancer stemness and activating lipid metabolism in PCa cells 16. In brief, all the abovementioned studies support a crucial role for Hippo-YAP signaling in modulating PCSC properties.

In the present study, we employed gain- and loss-of-function experiments to investigate the role of DCLK1 in maintaining the CSC properties of PCa cells and to elucidate whether DCLK1 exerts its effects via the Hippo pathway. Collectively, our results support the idea that overexpression of DCLK1 promotes the acquisition of a stem cell-like phenotype by inducing large tumor suppressor homolog 1 (LATS1)-mediated YAP signaling activation, ultimately leading to tumor growth in PCa.

## Materials and Methods

### Cell lines and cell culture

The human PCa cell lines 22Rv1 and PC3 were purchased from the Institute of Biochemistry and Cell Biology of the Chinese Academy of Sciences (Shanghai, China) and were authenticated by short tandem repeat profiling. Both cell lines were cultured in Dulbecco's modified Eagle's medium (Thermo Fisher Scientific, Waltham, MA, USA) supplemented with 10% fetal bovine serum, 100 U/ml penicillin and 100 μg/ml streptomycin in a humidified incubator containing 5% CO_2_ at 37 °C. The cells were screened monthly for mycoplasma contamination using the LookOut Mycoplasma PCR Detection Kit (Sigma‒Aldrich, St. Louis, MO, USA) and maintained for no longer than 6 months after resuscitation.

### Gene expression manipulations

Stable knockdown of DCLK1 in cells was achieved by lentivirus-mediated RNA interference using validated short hairpin RNA (shRNA) oligonucleotides inserted into the pLKO.1-puro vector (Obio Technology Corp. Ltd., Shanghai, China). The shRNA sequences are listed in Supplementary Table S1. To generate DCLK1-overexpressing PCa cell clones, the full-length cDNA encoding human DCLK1 was amplified by PCR and inserted into a lentiviral vector (Obio). The Hippo reporter constructs HOP-Flash (8x wild-type (wt) TEAD binding sites) and HIP-Flash (mutated TEAD binding sites) were obtained from Addgene (# 83467 and #83466; Addgene, Watertown, MA, USA). Transfection of plasmids into PCa cell lines was conducted using Lipofectamine 3000 (Thermo Fisher Scientific) according to the manufacturer's protocol. Cell lines with stable overexpression or silencing of DCLK1 were generated by lentiviral infection and selection for 10 days with 0.5 μg/ml puromycin.

### Western blotting

Total protein was extracted using radioimmunoprecipitation assay (RIPA) lysis buffer (Cell Signaling Technology, Danvers, MA, USA) supplemented with protease and phosphatase inhibitors following the manufacturer's instructions. Nuclear extracts were prepared using the Nuclear Extraction Kit (Sigma‒Aldrich) according to the suggested protocols. After protein quantification by the Bradford method with the BCA Protein Assay Kit (Thermo Fisher Scientific), equal quantities of proteins were separated by sodium dodecyl sulfate (SDS)-polyacrylamide gel electrophoresis and then transferred onto polyvinylidene fluoride membranes (Millipore, Billerica, MA, USA). After blocking with 5% nonfat milk for 60 minutes, the membranes were incubated overnight at 4 °C with the following primary antibodies: rabbit anti-DCLK1 (#AP7219b; Abgent, San Diego, CA, USA), rabbit anti-LATS1 (#3477; Cell Signaling Technology), rabbit anti-p-LATS1 (T1079) (#8654; Cell Signaling Technology), mouse anti-YAP (#12395; Cell Signaling Technology), and rabbit anti-p-YAP (S127) (#13008; Cell Signaling Technology). The blotted membranes were then stripped and reprobed with an anti-α-tubulin antibody (#T6199; Sigma‒Aldrich) as a loading control. Nuclear matrix protein P84 (#ab487; Abcam, Cambridge, MA, USA) was used as a nuclear marker. The protein bands were finally visualized using a FluorChem M system (ProteinSimple, San Jose, CA, USA), and the gray values were analyzed using ImageJ software (NIH, Bethesda, MA, USA).

### Colony formation assay

PCa cells were seeded into 6-well plates at a density of 1,000 cells per well and cultured for 10-14 days, after which the colonies were fixed with formaldehyde and stained with crystal violet. For quantitative analysis, the colonies containing more than 40 cells were counted and photographed. Each assay was independently repeated in triplicate.

### Sphere formation assay

A single-cell suspension of PCa cells was seeded at a density of 500 cells/well into 6-well ultralow attachment plates (Corning, Corning, NY, USA). The cells were cultured in serum-free DMEM/F12 medium (Thermo Fisher Scientific) supplemented with 2% B27, 20 ng/ml bFGF, 20 ng/ml EGF, 5 μg/ml insulin, and 0.4% BSA. After 10-12 days of culture, the number of spheres was determined, and images of the spheres were obtained under an inverted microscope. Each experiment was repeated at least 3 times.

### RNA extraction and quantitative reverse transcription-polymerase chain reaction (qRT‒PCR)

Total RNA was extracted from cultured cells using TRIzol reagent (Thermo Fisher Scientific) and reverse transcribed to cDNA using M-MLV Reverse Transcriptase (Promega, Madison, WI, USA) following the manufacturer's instructions. qRT‒PCR analysis was conducted using a SYBR Green I Master Kit (Roche, Mannheim, Germany) on a LightCycler® 480 System (Roche). Target gene expression levels were normalized to those of the housekeeping gene glyceraldehyde-3-phosphate dehydrogenase (GAPDH). The sequences of the primers used are listed in Supplementary Table S2. Relative gene expression levels were calculated as follows: 2^-[(Ct of gene) - (Ct of GAPDH)]^, where Ct is the threshold cycle value for the indicated transcript.

### Bioinformatics analysis

The correlation between DCLK1 expression and YAP1 expression, after normalization of each to GAPDH expression, in PCa was explored via the Gene Expression Profiling Interactive Analysis (GEPIA2) server (http://gepia2.cancer-pku.cn/#correlation) 17. A set of transcriptomic data for patients with prostate adenocarcinoma (n=498) was downloaded from The Cancer Genome Atlas (TCGA) in August 2022 and used to analyze the correlation between the mRNA expression level of DCLK1 and those of Hippo pathway genes. The 25th and 75th percentiles of DCLK1 expression were used as the cutoff criteria to classify the samples into the low (n= 125) and high (n= 125) DCLK1 expression groups. Differential expression analysis was performed using the R package 'Limma'.

### Immunofluorescence staining

PCa cells were seeded on coverslips in a 12-well plate at a density of 5 × 10^4^ cells/well. After reaching 70% confluence, the cells were fixed with 4% paraformaldehyde for 15 min and permeabilized with phosphate-buffered saline containing 0.5% Triton X-100 for 30 min. Following incubation with an Alexa Fluor^®^ 647-conjugated rabbit anti-YAP antibody (#38707; Cell Signaling Technology) at 4 °C overnight, the cells were counterstained with ProLong^®^ Gold Antifade Reagent with DAPI (#8961; Cell Signaling Technology) to visualize nuclei. Images of immunofluorescence staining were acquired using a confocal laser scanning microscope (LSM 710; Zeiss, Oberkochen, Germany).

### Luciferase activity assay

YAP/TAZ-TEAD transcriptional activity was assessed using HOP/HIP-flash luciferase reporter assays. In brief, PCa cells (3 × 10^3^ cells/well) were cultured in 48-well plates and allowed to adhere for 24 h. Then, the cells were cotransfected with 100 ng of a luciferase reporter plasmid or the control luciferase plasmid plus 5 ng of the pRL-TK Renilla plasmid (Promega) using Lipofectamine 3000 reagent (Thermo Fisher Scientific). Firefly luciferase and Renilla luciferase signals were measured 24 h after transfection using the Dual Luciferase Reporter Assay Kit (Promega) following the manufacturer's instructions. Reporter activity was normalized to Renilla luciferase activity as the internal control.

### *In vivo* animal studies

All animal maintenance and experimental procedures were conducted under a protocol approved by the Institutional Animal Care and Use Committee of Sun Yat-sen University. Male BALB/c nude mice (18-20 g) aged 5 weeks were obtained from SLAC Jingda Laboratory Animal Company (Changsha, China). Mice were randomly allocated into three groups (n = 5 mice per group), namely, the vector control group, the DCLK1-overexpressing group, and the verteporfin treatment group for assessment of tumorigenic potential. The indicated 22Rv1 cells (1 × 10^5^) in Matrigel were inoculated subcutaneously into the dorsal flank area of each mouse. Then, to verify the regulatory effect of DCLK1 on Hippo signaling *in vivo*, verteporfin (100 mg/kg body weight) was injected intraperitoneally into mice in the DCLK1-overexpressing group every 2 days 18, 19. Tumor volume was measured every 5 days using an external caliper and calculated as follows: V = (length × width^2^)/2. After the animals were sacrificed, the tumors were excised, weighed and fixed in formalin for pathologic examination.

### Immunohistochemistry and scoring

Formaldehyde-fixed and paraffin-embedded tumor tissues were sliced into 4-μm-thick sections. Then, the sections were deparaffinized and rehydrated through an ethanol gradient prior to antigen retrieval in citrate buffer. Thereafter, the sections were incubated with primary antibodies, including rabbit anti-DCLK1 (#AP7219b; Abgent), rabbit anti-CD44 (#ab157107; Abcam), and anti-NANOG antibody (#ab109250; Abcam), at 4 °C overnight. The sections were then incubated with secondary antibodies. Protein expression was semiquantitatively scored as described in our previous report 10, as follows: the staining intensity was scored as 0 (no staining), 1 (weak staining), 2 (moderate staining), or 3 (strong staining), and this score was multiplied by the percentage of cells with each staining intensity score, resulting in a total IHC score ranging from 0-300.

### Statistical analysis

Statistical analyses were conducted using IBM SPSS Statistics (version 20.0). The data are presented as the mean ± standard error of the mean (SEM) of data from at least three separate experiments and were compared using Student's t test. All significance tests were two-tailed, and *P* < 0.05 was considered to indicate statistical significance.

## Results

### DCLK1 promotes the stem cell-like phenotype of PCa cells

**Figure 1:**
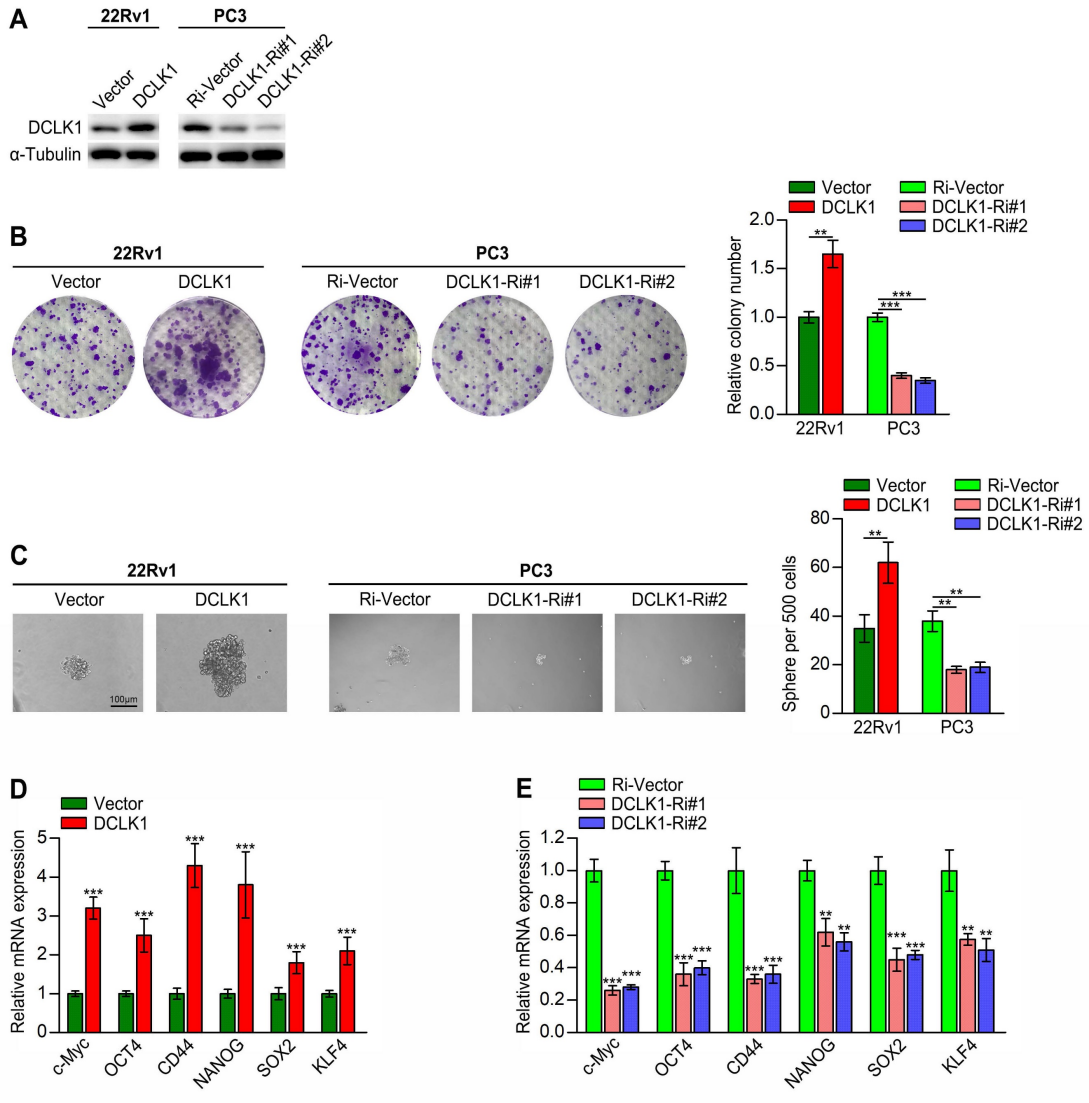
DCLK1 promotes the stem cell-like phenotype of PCa cells. (**A**) Western blot analysis confirmed the DCLK1 expression level in 22Rv1 and PC3 cells after transduction. (**B**) Representative images (left panel) and comparative quantification (right panel) of colonies formed by the indicated cell lines. (**C**) Representative light micrographs (left panel) and comparative quantification (right panel) of the sphere formation capacity of the indicated cell lines. (**D** and **E**) Relative mRNA expression levels of stemness-related markers and regulators in the established PCa cell lines, as determined by qRT‒PCR. The data are presented as the means ± SDs from three independent experiments, and *P* values were determined by two-tailed Student's t test; **P < 0.001, ***P < 0.001.

There is compelling evidence that the acquisition of a CSC-like phenotype is essential for self-renewal, tumorigenesis, metastasis and drug resistance in PCa 3, 4, 20. Here, we employed gain- and loss-of-function studies to investigate the role of DCLK1 in maintaining the CSC properties of PCa cells. We have verified in our previous research that DCLK1 mRNA and protein expression were remarkably different in a series of PCa cell lines (LNCaP, PC3, DU145, and 22Rv1), with the highest expression level in PC3 and lowest in 22Rv1 10. Hence, 22Rv1 cells with DCLK1 overexpression and PC3 cells with silencing of DCLK1 were engineered via lentiviral transduction (Figure 1A). First, we conducted *in vitro* assays, which demonstrated that compared to the corresponding vector control cells, DCLK1-overexpressing 22Rv1 cells formed more and larger colonies, whereas DCLK1-knockdown PC3 cells formed markedly fewer and smaller colonies (Figure 1B). These findings indicated that DCLK1 may play a crucial role in the self-renewal of PCa cells. In addition, the tumor sphere formation assay is a well-established method for evaluating the self-renewal capability of stem cells. Herein, the sphere formation assays revealed that upregulating DCLK1 increased but downregulating DCLK1 decreased the size and number of tumor spheroids formed from 22Rv1 and PC3 cells (Figure 1C). Moreover, the expression of a series of stemness-related markers and regulators, including c-Myc, OCT4, CD44, NANOG, SOX2, and KLF4, was measured by qRT‒PCR in the established PCa cell lines. The results demonstrated notably higher mRNA expression levels of these factors in DCLK1-overexpressing cells but significantly lower levels in DCLK1-knockdown cells than in the corresponding control cells (Figure 1D-E). Collectively, these results reveal that DCLK1 promotes a stem cell-like phenotype in PCa cells.

### Bioinformatics analysis revealed a positive correlation between DCLK1 and the Hippo pathway

**Figure 2:**
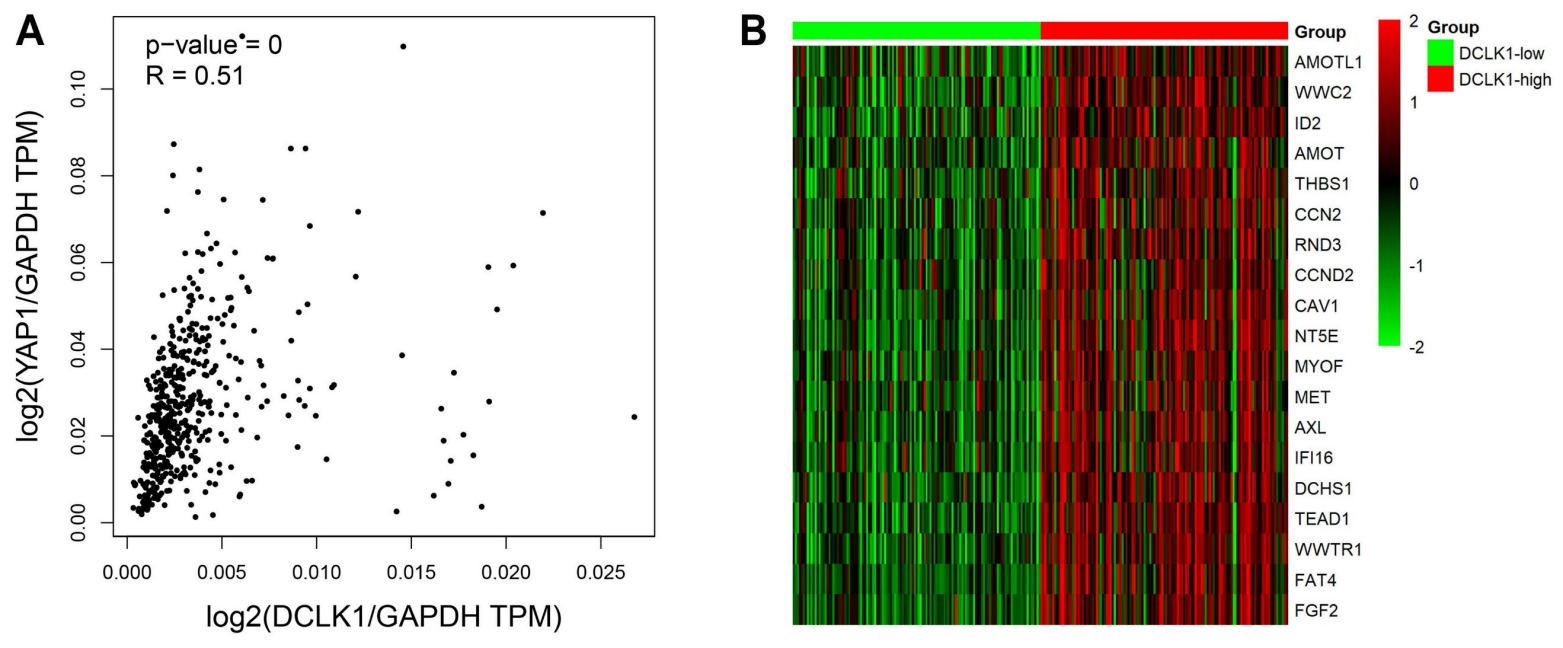
Bioinformatics analysis revealed a correlation between DCLK1 and the Hippo pathway. (**A**) The correlation between DCLK1 expression and YAP1 expression was explored via the GEPIA2 server. The scatter plot shows that there was a significant positive correlation between the expression levels of DCLK1 and YAP1 in PCa cells (both levels were normalized to that of GAPDH). TPM, transcripts per million. (**B**) The mRNA expression profiles of 498 PCa samples in TCGA were evaluated. The heatmap shows that there was a strong correlation between the mRNA expression levels of DCLK1 and representative Hippo signaling pathway genes. The red text represents a gene with a high rank, while the green rectangle indicates decreased gene expression in tumor samples. The 25th and 75th percentiles of DCLK1 expression were used to classify samples into the low (n= 125) and high (n= 125) DCLK1 expression groups.

DCLK1 expression has been shown to be upregulated in multiple human cancers, including breast cancer, colorectal cancer and pancreatic cancer 5, 6, 8. Accumulating evidence supports the idea that DCLK1 is essential for tumorigenesis, metastasis and the maintenance of cancer stemness traits, consistent with our previous research on PCa 9, 10. Moreover, compelling data now support the crucial role of the Hippo pathway in modulating PCa initiation, progression, and stem cell characteristics 13-16. Interestingly, a recent molecular study revealed that inhibition of DCLK1 could downregulate PD-L1 expression through the Hippo-YAP1 signaling pathway in human pancreatic cancer 21. We thus suggested the intriguing possibility that DCLK1 may be correlated with the Hippo pathway in PCa. To investigate this possibility, we evaluated the changes in the mRNA expression profiles of components of the Hippo signaling pathway in TCGA datasets. We discovered a significant positive correlation between the expression level (transcripts per million, TPM) of DCLK1 and that of YAP1 in PCa (P < 0.001, R = 0.51) (Figure 2A). Besides, we have also used the GEPIA2 database to explore the correlation between DCLK1 and key target genes of several signaling pathways which have been tested for DCLK1 involvement in stem cell-like traits in other cancers. The results demonstrated that DCLK1 was positively correlated with all these key target genes, and the correlation coefficient R with the Hippo pathway (YAP1, R = 0.51) was higher than with NOTCH pathway (Hes1, R = 0.37; Hes3, R = 0.25; Hes5, R = 0.22), WNT pathway (CCND1, R = 0.16; c-Myc, R = 0.27; CTGF, R = 0.24), and Hedgehog pathway (GLI1, R = 0.33; GLI2, R = 0.41; GLI3, R = 0.35). Furthermore, analysis of data from 498 TCGA PCa samples revealed that there were strong positive correlations between the mRNA expression level of DCLK1 and those of Hippo signaling pathway components, including CTGF, AREG, BIRC5, AMOTL1, and TEAD1 (Figure 2B). Thus, these results indicate that DCLK1 is closely correlated with the Hippo signaling pathway in PCa.

### DCLK1 inhibits the Hippo pathway, leading to YAP activation via LATS1

**Figure 3:**
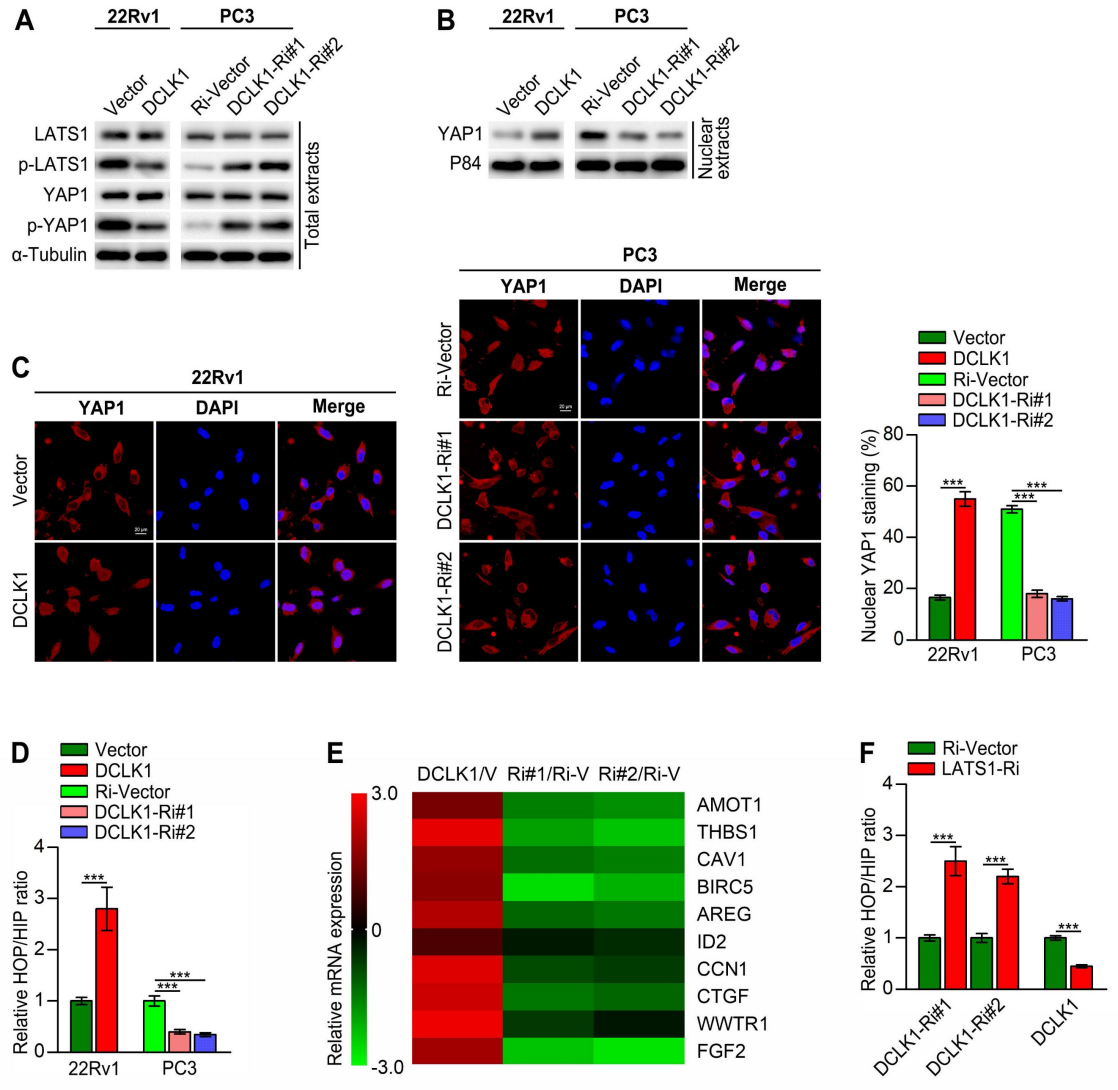
DCLK1 inhibits the Hippo pathway, leading to YAP activation. (**A** and **B**) Western blot analysis of the expression of the indicated proteins in the indicated cells. α-Tubulin was used as a loading control for total extracts, and P84 was used as a loading control for nuclear extracts. (**C**) The expression and subcellular localization of YAP1 were evaluated in the indicated cells with altered DCLK1 expression and visualized by fluorescence and laser confocal microscopy (left panel). Scale bars: 20 μm. Percentage of nuclear YAP1 in the indicated cells with altered DCLK1 expression (right panel). (**D**) HOP/HIP luciferase reporter activity, indicating YAP/TAZ-TEAD transcriptional activity, was measured in the indicated cells. (**E**) Log2-transformed fold changes in the mRNA expression levels of the indicated genes, as determined by qRT‒PCR analysis comparing DCLK1-overexpressing cells versus vector cells (V) or DCLK1-Ri (Ri#1 and Ri#2) cells versus Ri-vector (Ri-V) cells. (**F**) YAP/TAZ-TEAD reporter activity was abolished by downregulation of LATS1 in the indicated cells. The data are presented as the means ± SDs from three independent experiments, and *P* values were determined by two-tailed Student's t test; ***P < 0.001.

To further elucidate the correlation between DCLK1 and the Hippo pathway in PCa, we then conducted a series of *in vitro* experiments. Western blot analysis revealed that DCLK1 overexpression decreased the levels of phosphorylated LATS1 and YAP1 and increased the nuclear abundance of YAP1 in 22Rv1 cells, whereas DCLK1 knockdown had the opposite effects on PC3 cells (Figure 3A-B). Moreover, immunofluorescence staining revealed that DCLK1 overexpression promoted the nuclear translocation of YAP1 in 22Rv1 cells, while nuclear YAP1 staining decreased following inhibition of DCLK1 expression in PC3 cells (Figure 3C). YAP is a transcriptional coactivator that can shuttle from the cytoplasm to the nucleus, where it functions principally by binding to members of the TEA domain (TEAD) family of transcription factors together with another coactivator, transcriptional coactivator with PDZ-binding motif (TAZ).^11^ Hence, HOP/HIP-flash luciferase assays were carried out to further explore the effect of DCLK1 on YAP/TAZ-TEAD transcriptional activity. As predicted, the HOP/HIP activity ratio was markedly elevated in DCLK1-overexpressing 22Rv1 cells and decreased in DCLK1-downregulated PC3 cells (Figure 3D). Accordingly, the relative mRNA expression of classical YAP/TAZ downstream target genes was significantly increased in DCLK1-upregulated cells but was impaired after knockdown of DCLK1 (Figure 3E). Typically, when the Hippo kinase module is “active”, LATS1/2 phosphorylate and thereby inhibit YAP and its paralog TAZ. To further verify whether LATS1/2 are involved in the DCLK1-mediated regulation of HOP/HIP reporter activity, we knocked down LATS1 in the established PCa cell lines. Notably, DCLK1 silencing-mediated inhibition of YAP/TAZ-TEAD reporter activity was abolished by knockdown of LATS1, and the same abolishing effect on YAP/TAZ-TEAD reporter activity was observed in DCLK1-overexpressing cells (Figure 3F). These results confirm that DCLK1 inhibits the Hippo signaling pathway, leading to YAP activation via LATS1 in PCa cells.

### DCLK1 promotes stemness traits in PCa through YAP signaling

**Figure 4:**
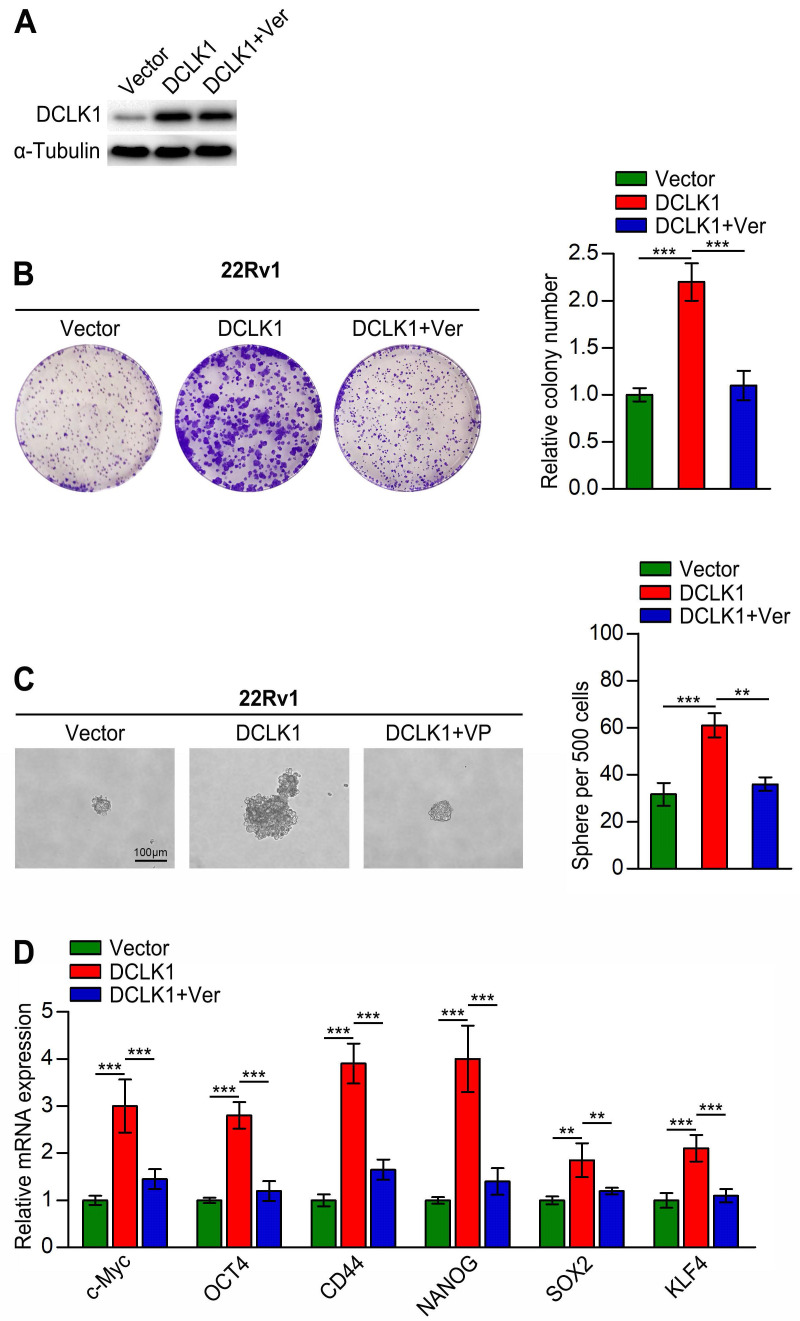
DCLK1 regulates stemness traits in prostate cancer cells through YAP signaling. (**A**) Western blot analysis confirmed the DCLK1 expression level in the indicated cells. (**B**) Representative images (left panel) and comparative quantification (right panel) of colonies formed by the indicated cell lines. (**C**) Representative light micrographs (left panel) and comparative quantification (right panel) of the sphere formation capacity of the indicated cell lines. (**D**) Relative mRNA expression of stemness-related markers and regulators analyzed by qRT‒PCR in the indicated cells. Ver, verteporfin (10 µM, 3 h). The data are presented as the means ± SDs from three independent experiments, and *P* values were determined by two-tailed Student's t test; **P < 0.001, ***P < 0.001.

Although we confirmed that DLCK1 inhibits the Hippo signaling pathway in PCa cells, whether this effect is related to cancer stemness traits is unknown. To explore whether DCLK1 promotes stemness traits in PCa through impacts on the Hippo signaling pathway, we treated DCLK1-overexpressing 22Rv1 cells with the YAP inhibitor verteporfin. As a small molecule inhibitor, verteporfin can prevent the interaction between YAP/TAZ and TEAD transcription factors, but it only weakly affected the expression level of DCLK1 (Figure 4A). The results of *in vitro* experiments showed that verteporfin treatment significantly reduced the colony size and number of DCLK1-overexpressing 22Rv1 cells, and a similar abolishing effect was observed in the tumor sphere formation assay (Figure 4B-C). Moreover, the relative mRNA expression levels of stemness-related markers and regulators were dramatically decreased after treatment with verteporfin (Figure 4D). Collectively, these results reveal that DCLK1 relies on YAP signaling to modulate the stemness properties of PCa cells.

### DCLK1 promotes the tumorigenicity and stem cell-like traits of PCa cells *in vivo*

**Figure 5:**
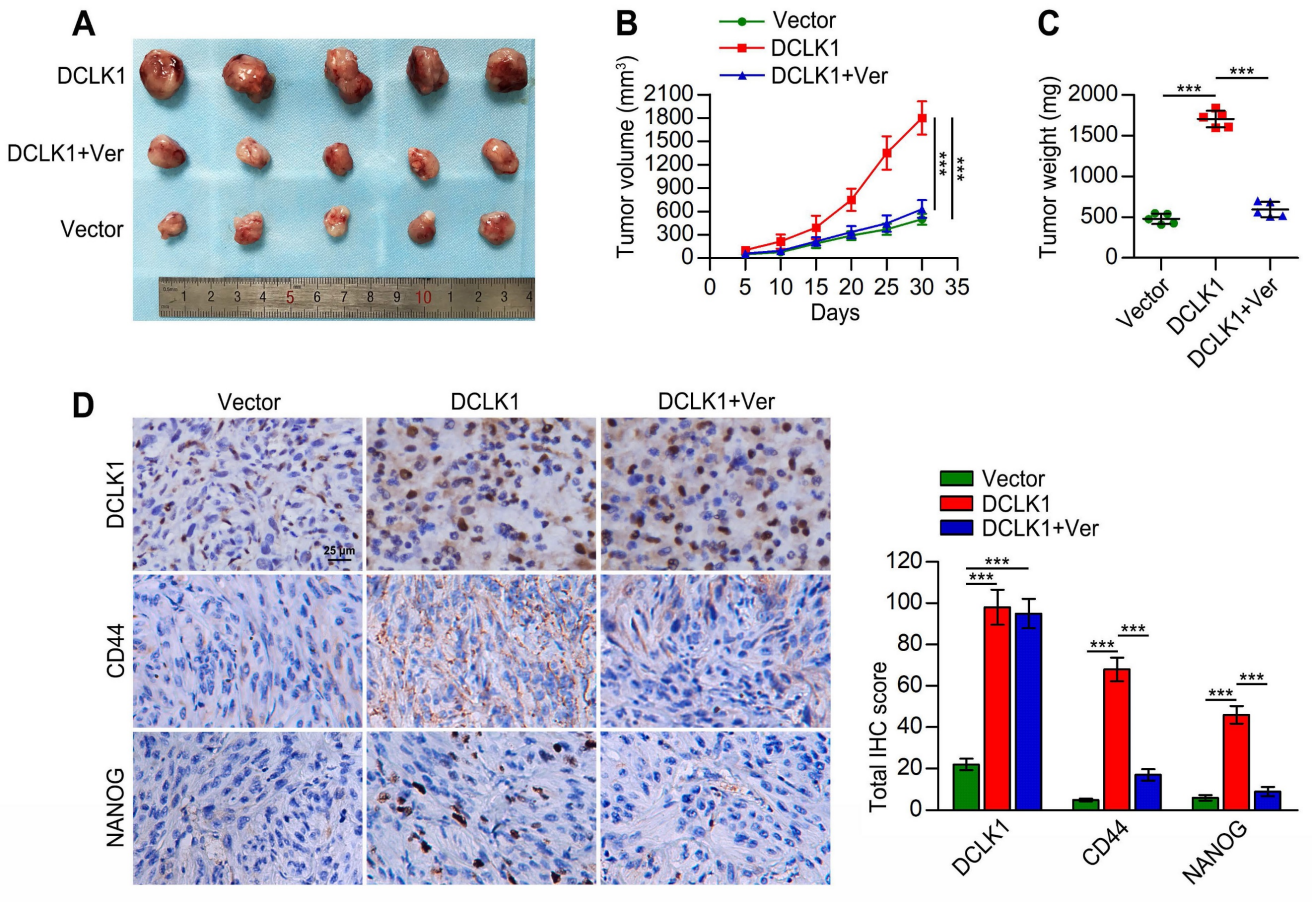
DCLK1 promotes PCa tumorigenicity *in vivo*. (**A**) Images of tumors formed in nude mice injected with the indicated cell lines. (**B**) Growth curves of the indicated xenograft tumors (n = 5 mice/group) during the experimental period. (**C**) Xenograft tumor weights at the end of the experimental period. (**D**) Representative images (left panel) and comparative quantification (right panel) of immunohistochemical staining for DCLK1, CD44, and NANOG in the indicated groups. Ver, verteporfin (100 mg/kg body weight, every 2 days, intraperitoneal injection). The data are presented as the means ± SDs from three independent experiments, and *P* values were determined by two-tailed Student's t test; ***P < 0.001.

Considering the involvement of DCLK1 in PCa tumorigenesis and progression and its potential role in promoting the stem cell-like properties of PCa cells via Hippo signaling, as demonstrated above, we further explored whether these observations could be reproduced *in vivo*. As shown in Figure 5A-C, the tumors formed by DCLK1-overexpressing 22Rv1 cells were markedly larger and heavier than the vector control tumors. Regarding the effect of verteporfin in tumor cell proliferation, Nguyen *et al.* 22 found that verteporfin treatment did not have a statistically significant impact on the growth of pre-established orthotopic PC3 xenografts. Similarly, the tumor size and endpoint measurement of tumor weight of subcutaneous SB1 tumors were not reduced with the treatment of verteporfin in the animal experiments conducted by Fu *et al.* 23. In our study, consistent with the results of the *in vitro* assays, dramatically reduced volumes and weights of DCLK1-overexpressing 22Rv1 xenograft tumors were observed after verteporfin treatment. Furthermore, we measured the expression of DCLK1 and the stemness-related markers CD44 and NANOG in tumor tissues from the mice described above by immunohistochemical (IHC) staining (Figure 5D). The expression levels of DCLK1, CD44 and NANOG in DCLK1-overexpressing 22Rv1 xenograft tumors were significantly higher than those in the vector control xenograft tumors. In addition, the administration of verteporfin did not appear to affect DCLK1 expression but markedly decreased the expression of CD44 and NANOG. Thus, these results confirm that DCLK1 plays a critical role in PCa tumorigenesis and the maintenance of the stem cell-like traits of PCa cells via Hippo signaling *in vivo*.

### Hypothetical model of DLCK1-mediated signaling revealed by this study

**Figure 6:**
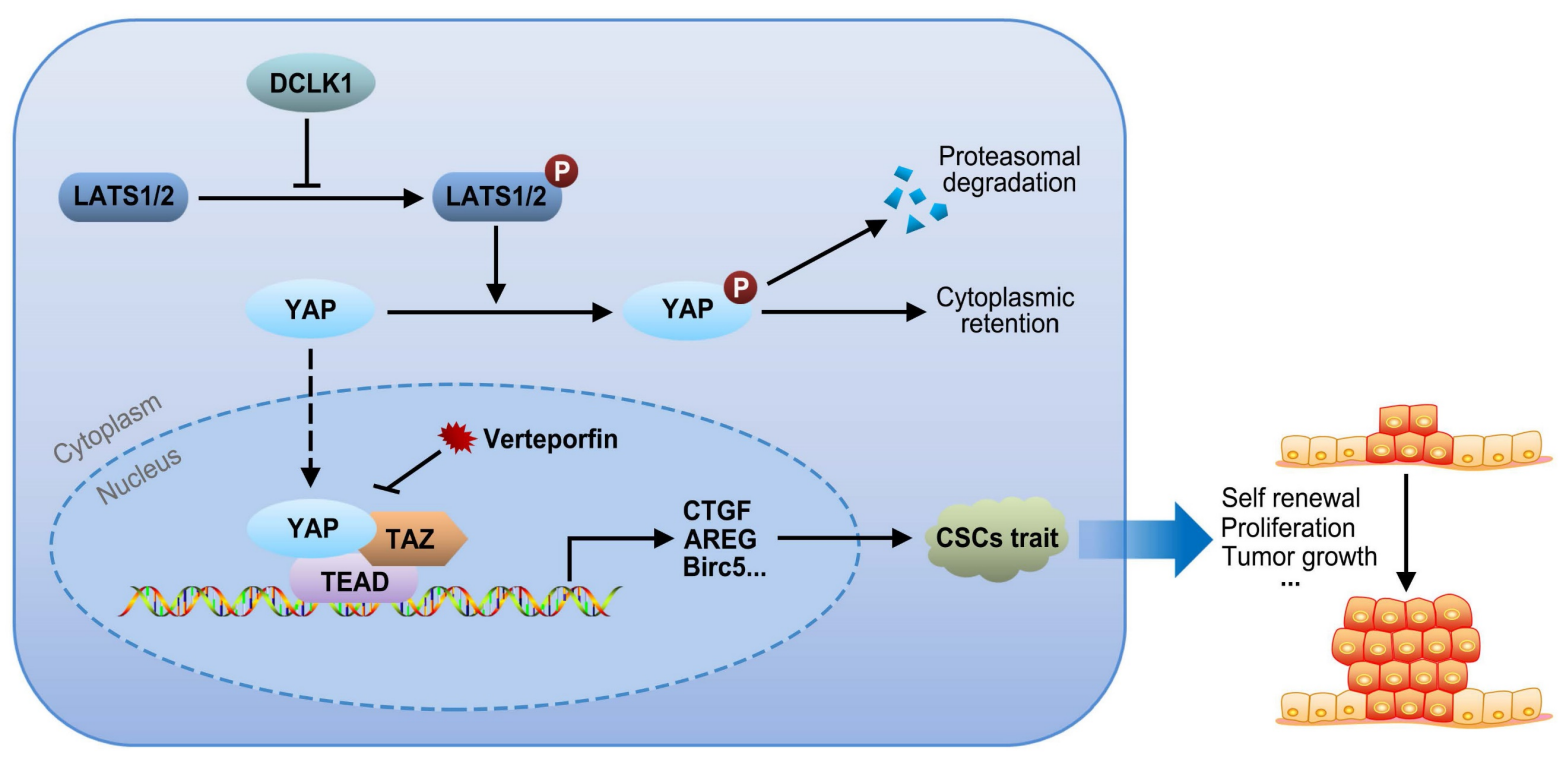
Signaling pathway model. Overexpression of DCLK1 promotes a stem cell-like phenotype by inducing LATS1-mediated YAP signaling activation, ultimately leading to tumor growth and progression in PCa.

The core Hippo-YAP pathway components constitute a nuclear transcriptional module and a cytoplasmic kinase module. The major function of the kinase cascade is to inhibit the oncogenic transcriptional cascade containing YAP1. In the canonical pathway, cytoplasmic YAP1 is phosphorylated in response to upstream phosphorylation and activation of LATS1 and LATS2, leading to cytoplasmic retention or subsequent ubiquitination-mediated proteasomal degradation of YAP1. Alternatively, when LATS1/2 are inactivated due to the upregulation of DCLK1, the nuclear translocation of cytoplasmic YAP1 is increased, and the expression of target genes involved in CSC traits, such as self-renewal, proliferation and tumor growth, is consequently increased via interactions of YAP1 with TEADs and TAZ. Collectively, our results support the idea that overexpression of DCLK1 promotes a stem cell-like phenotype by inducing LATS1-mediated YAP signaling activation, ultimately leading to tumor growth and progression in PCa (Figure 6).

## Discussion

Over the past few decades, tumor-targeted therapies have been extensively investigated and developed based on an in-depth understanding of cancer biology. Despite the recent advances in targeted therapy and immunotherapy, androgen-resistant and metastatic PCa remain refractory to treatment and the leading cause of patient mortality 24. Mechanisms underlying the progression of lethal PCa are far from being completely understood, and revealing the molecular basis of PCa aggressiveness to develop novel intervention strategies is of crucial importance. The presence of CSCs provides a theoretical explanation for the resistance of advanced PCa to the abovementioned treatment options 20, 25. In this study, we found that upregulating DCLK1 promoted but downregulating DCLK1 suppressed the PCa stem cell-like phenotype, and further *in vitro* and *in vivo* experiments confirmed that DCLK1 promoted the tumorigenicity and stem cell-like properties of PCa cells via Hippo-YAP signaling. Our findings shed light on new potential diagnostic and therapeutic targets for advanced PCa.

A specialized subset of tumor cells that harbor unique stemness traits, such as tumor-initiating potential and self-renewal capacity, CSCs have been proposed to be responsible for metastasis, recurrence and drug resistance, which are the major causes of cancer mortality 3. Hence, exploring CSC-specific signaling characteristics and mechanisms is clinically important for improving tumor-targeted therapy. Like other CSCs, PCSCs are one of the major emerging explanations for the refractoriness of PCa to conventional treatments and are closely involved in PCa initiation, growth, metastasis, relapse and chemoresistance 3, 4. Recent *in vitro* studies demonstrated that PCSCs could be enriched after hormone deprivation or docetaxel treatment and that this enrichment might be responsible for chemoresistance, recurrence and poor outcomes in patients with advanced PCa treated with the abovementioned strategies 3, 4, 20. However, the molecular mechanism and signaling pathway underlying the regulation of CSCs and their stemness traits in PCa remain undetermined. Therefore, the successful identification of potential critical markers that govern the stemness of PCSCs holds promise for improving the outcomes of PCa patients.

As a well-recognized marker for CSCs in colon, pancreatic, gastrointestinal, breast, and esophageal cancers, DCLK1 is linked to more aggressive tumor types and treatment resistance 9. Regarding the human prostate, IHC staining of tissue microarrays across human cancers revealed minimal or no DCLK1 expression in the normal prostate but strong immunoreactivity of DCLK1 in the cytoplasm of PCa cells 5, consistent with the results of the cellular immunofluorescence analysis performed by Vlajic *et al.* 26. In addition, Roudier *et al.* 27 reported that the protein level of DCLK1 was greater in unpaired CRPC metastases tissues than in primary PCa tissues and that compared with DCLK1- patients, DCLK1+ patients exhibited a shorter time to biochemical recurrence, suggesting that DCLK1 plays a crucial role in the progression of primary PCa to metastatic CPRC. In a recent prospective cohort study, next-generation RNA sequencing analysis and Ingenuity Pathway Analysis revealed that DCLK1 was upregulated after 4 weeks of ADT in patients with localized PCa 28. Moreover, in exploring the role of resveratrol as a radiosensitizer by targeting CSCs among radioresistant PCa cells (PC-3), El-Benhawy *et al.* 29 found that DCLK1 expression, as measured by qRT‒PCR, was significantly decreased following treatment with ionizing radiation. Collectively, all the important evidence mentioned above, together with our preceding findings 10, support the crucial role of DCLK1 and CSCs in promoting PCa aggressiveness. However, the underlying correlation between DCLK1 and CSCs in PCa remains unclear.

Several lines of evidence demonstrated in the current study indicate that DCLK1 enhances PCSC properties: (1) the serial colony formation and sphere formation capacities of PCa cells were significantly increased by forced expression of DCLK1; (2) the expression of the stemness signature genes c-Myc, OCT4, CD44, NANOG, SOX2, and KLF4 increased following DCLK1 overexpression in PCa cells; and (3) the limiting dilution xenograft tumor formation assay showed that overexpression of endogenous DCLK1 markedly increased the tumor size and the expression of CD44 and NANOG, both of which were attenuated after verteporfin treatment. Taken together, these results reveal that upregulation of DCLK1 indicates greater biological aggressiveness and may be critical factor underlying PCa progression through pathways linked to CSCs.

Although we revealed that DCLK1 promotes the stem cell-like phenotype of PCa cells, the underlying molecular mechanisms remain unclear. Importantly, accumulating evidence supports the crucial role of Hippo-YAP signaling in modulating tumor initiation, tumor progression, and stem cell-like properties in PCa 13-16. Regarding the relationship between DCLK1 and the Hippo pathway, Chen *et al.* 30 reported that DCLK1 modulated the type II-to-type I differentiation of alveolar epithelial cells (AECs) via the Hippo pathway and promoted stemness in type II AECs (AECIIs), possibly through the inhibition of LATS1/2 activation, leading to the activation of YAP by its dephosphorylation. Similarly, a molecular study conducted by Yan *et al.* 21 showed that upregulation of DCLK1 promoted PD-L1 expression through the Hippo-YAP1 pathway in human pancreatic cancer. Moreover, Yuliani *et al.* 31 reported that thrombin induced IL-8/CXCL8 expression via DCLK1-dependent YAP activation in human lung epithelial cells. Despite the crucial role of YAP1 in regulating these physiological processes, the biological significance of the interaction between DCLK1 and YAP1 in PCa remains incompletely elucidated.

From a mechanistic perspective, through TCGA database analysis, we discovered that DCLK1 expression in PCa is closely correlated with many critical molecules of the Hippo pathway. Although bioinformatics analysis indicated a positive correlation between the mRNA expression of DCLK1 and YAP1 in tumor samples (Figure 2A), there were no significant changes in the mRNA or protein expression levels of YAP1 in PCa cell lines after DCLK1 overexpression or knockdown at the cellular level (Figure 3A). The reason for the inconsistency may be due to the different biological characteristics and microenvironments of clinical tumor samples and *in vitro* cell lines. However, the abovementioned inconsistency will not affect the logicality and reliability of our research conclusions. Canonically, the Hippo core cascade is carcinostatic and functions by impairing the nuclear translocation of YAP1 and promoting its proteasome-mediated degradation in the cytoplasm 11. In brief, YAP1 is phosphorylated at Ser127 by upstream activation of Lats1/2 and then accumulates in the cytoplasm, where it interacts with 14-3-3 proteins and is degraded. In contrast, dysregulation of Hippo-YAP signaling results in reduced phosphorylation of YAP1 but promotion of its translocation into the nucleus, where it interacts with its transcriptional coactivator TAZ, leading to the transcription of downstream target genes involved in tumorigenesis and aggressive phenotypes 32. Consistent with the above defined molecular mechanisms, our further investigations confirmed that DCLK1 reduced the levels of phosphorylated LATS1 and YAP1, leading to the nuclear translocation of YAP1 and a subsequent increase in HOP/HIP reporter activity, which was abolished by inhibition of LATS1. Dysregulation of the Hippo pathway is implicated in many types of cancers with downregulated LATS1/2 signaling, resulting in increased accumulation of active YAP1 in the nucleus 33-36. However, disruption of YAP1 signaling in the nucleus can be achieved by the porphyrin compound verteporfin, which is a photosensitizer used to treat excess vascularization in eye disease and has been found to bind to YAP1 and change its conformation to inhibit the YAP1/TEAD interaction (Figure 6) 18, 37. Considering this observation, we carried out *in vitro* assays using verteporfin and demonstrated that the ability of DCLK1 to promote the stemness phenotypes of PCa cells relies on Hippo-YAP signaling.

This study has several limitations. As a serine/threonine kinase, DCLK1 has been shown to phosphorylate target proteins such as synapsin II and myelin basic protein, and its kinase activity has also been shown to negatively modulate tubulin polymerization 38. Thus, the role of DCLK1 kinase activity toward different proteins appears inconsistent 39. Herein, we found that DCLK1 promoted YAP signaling in a manner dependent on LATS1 activation in PCa cells. However, the details regarding the mechanism by which DCLK1 regulates LATS1 remain unclear, and future investigations are warranted to explore whether DCLK1 plays a role in the activation of LATS1 via phosphorylation. Technically, 22Rv1 is considered to be androgen-independent and AR positive, and PC3 cells are classified as androgen-independent PCa cells with lack of AR/PSA expression. As different phenotypes of PCa cell lines, like androgen responsive or independent, positive or lack of AR/PSA expression, and origins, may result in diverse association between DCLK1 and stemness traits in PCa cell lines. Our group will explore the correlation of DCLK1 with androgen and AR during our future studies, aiming to better elucidate the impact of DCLK1 on PCa aggressiveness. Furthermore, our study lacks strong evidence supported by clinical data. Hence, future validation experiments based on human PCa tissues or organoids are necessary.

Taken together, the data presented above support the speculation that overexpression of DCLK1 promotes stem cell-like characteristics by inducing LATS1-mediated YAP signaling activation, ultimately leading to tumor growth and progression in PCa. More crucially, our findings provide new insight for clarifying the potential mechanisms of PCa progression and indicate that DCLK1 may be a therapeutic target for PCa.

## Supplementary Material

Supplementary tables.
