# Supplementary material for: Doublecortin-like kinase 1 promotes stem cell-like properties through the Hippo-YAP pathway in prostate cancer
Source: Int J Med Sci. 2025 Jan 1;22(2):460–72. doi: 10.7150/ijms.99062 (PMC11704687; doi:10.7150/ijms.99062)
Supplement: Supplementary file 1 — Supplementary tables. [file ijmsv22p0460s1.pdf]

Supplementary Information

Table S1 The shRNA sequences for knockdown of DCLK1

shRNA		Sequence (5'-3')
**sh-DCLK1 #1**	sense	GATCCGCCACCATGTCAGTGTCTTCAAGAGACAG ACGACTCATGGTGCTTTTT
	anti-sense	AATTCAAAAAACTCATGGTGCTGACAGTGTCTTCT CTTGAAAGACAGACACTCATGGTG
**sh-DCLK1 #2**	sense	GATCCGCACATCTTCAAAGTGTCTTCAAGAGACA GACACTTTGAATGATGTTTTT
	anti-sense	AATTCAAAAAACATCTTCAAAGACAGACACTTTGA ATGATGTGTGCTTTTT

Table S2 Primers for real-time PCR analysis

Gene		Sequence (5'-3')
**DCLK1**	forward	CAGCAACCAGGAATGTATTGGA
	reverse	CTCAACTCGGAATCGGAAGACT
**c-Myc**	forward	CAACTCGTTCGTGGTAGTGTGG
	reverse	TGGATGCTGCATGAGTGACCT
**OCT4**	forward	GGGGTGCGCTCTGTCTTTGC
	reverse	GGCATTTTCTTTCCCTCAGGACAG
**NANOG**	forward	CTGGAGCTCTTCAGCATTAC
	reverse	CAAATGTGATAGGTGATTTGT
**SOX2**	forward	GGAAGGAGAAGTGGGAGC
	reverse	CAGGCTGGGCTCTGTTT
**KLF4**	forward	GCCTCCTCCACCTCGTGTC
	reverse	GCTTGCCTTCCCCTTCTTC
**AMOT1**	forward	GGAGCCATCTTCTCTGCTC
	reverse	TTGCCTTCATCCTTTGCTC
**THBS1**	forward	GAGATGGAGGAGGAGGATGA
	reverse	CCAGTTTCTGACTTCACACCA
**CAV1**	forward	GGAGCAGGAAAGACAAAGG
	reverse	TTTGTAGGTGGATCTGCTC
**BIRC5**	forward	AGGACCACCGCATCTCTACAT
	reverse	AAGTCTGGCTCGTTCTCAGTG
**AREG**	forward	CTGGTGATGACACTGGAAAC

	reverse	CTCTGGTTTGCTGTCTTTGC
**ID2**	forward	GCAAATGTCTGGTGGATGGA
	reverse	GCTCAGATAGTTGAGGTGGTGAA
**CCN2**	forward	CAGCATGGACGTTCGTCTG
	reverse	AACCACGGTTTGGTCCTTGG
**CTGF**	forward	GCCTGGTGCTGAGGAAATGA
	reverse	CCAGTGTCTCCTTTCATCAGC
**WWTR1**	forward	GGACAGGAAGATGGTGGTGA
	reverse	CCAGTCCTTCTCCTCCTCC
**FGF2**	forward	AGCGCCTCTCTCGCATACAG
	reverse	CCAGCGTGTCCAGTTCTTCAG
**GAPDH**	forward	AAGGTGAAGGTCGGAGTCAA
	reverse	AATGAAGGGGTCATTGATGG
